# Reopening America's Schools During the COVID-19 Pandemic: Protecting Asian Students From Stigma and Discrimination

**DOI:** 10.3389/fsoc.2020.588936

**Published:** 2020-11-30

**Authors:** Daisuke Akiba

**Affiliations:** School of Education, Queens College and The Graduate Center of The City University of New York, Queens, NY, United States

**Keywords:** COVID-19, discrimination, Asian, Asian-American, pandemic, racism, school reopening, coronavirus

## Abstract

The COVID-19 outbreak has prompted a rise in stigma and discrimination against people of Asian descent in many areas in the world, including the United States^1^. Anti-Asian hate incidents, which have ranged from verbal attacks, refusal of service to physical assault, continue to transpire in the U.S., and they put psychological and physical well-being of Asian children at increased risk. Discussions toward reopening of U.S. schools thus far, however, seem to have exclusively included the infection-related concerns and pedagogical consequences of continued disruptions in face-to-face instructions. Hence, educators, policymakers, and other stakeholders need to have plans in place to ensure that reopening of schools will be a smooth experience for students of all backgrounds.

## Introduction

Well before the World Health Organization (WHO) declared COVID-19 a pandemic on March 11, 2020, with a presumed origin of the virus in Wuhan, China, stigma and hostility against Chinese people had already begun surfacing across the globe, and the victims included anybody who appeared Asian (Litam, 2020). Although the media have displayed a tendency to treat anti-Asian hate incidents as a rarity, thousands of these anti-Asian incidents have been reported (Zhou, 2020). These anti-Asian incidents range from refusal of service, peer harassment, xenophobic rants, to physical assault (Anti-Defamation League, 2020), with reportedly 6% of the cases involving children as victims (Leung and Nham, 2020). A surge in these anti-Asian incidents was reported in July, 2020 (Hsu, 2020), and anecdotes of Asians in the United States being victimized continue to pour in, as we approach the end of the year (Jiji.com, 2020).

Anti-Asian xenophobia has been witnessed, even in the most unexpected places. In January, 2020, students at the University of California, Berkeley found a communique from the University's Health Services, affirming that “*fears about interacting with those who might be from Asia*” was among the “*normal reactions*” to COVID-19—thereby normalizing the stigmatization of individuals that *might* be from Asia (Chiu, 2020). While the communication was later retracted, one may find it troubling that an educational institution would formally offer sympathetic endorsement of xenophobia against an entire ethnicity. Similarly, the U.S. Centers for Disease Control and Prevention (CDC) has featured Asian-themed graphics, such as Chinese food and an Asian tapestry, utterly out of context, alongside information on COVID-19—with no other cultures being represented in this fashion (Harris, 2020). While this may not have been a blatant display of anti-Asian xenophobia, one may find the implicit connection drawn by a government agency between the global pandemic and a specific culture disconcerting.

The climate of xenophobia against Asians—or *Asiaphobia*—continues to be prevalent in the U.S., and experiences of stigma and discrimination are likely to have an adverse psychological impact on them (Le et al., 2020; Misra et al., 2020). While empirical research has not been widely disseminated given the recency of the situation, Charissa Cheah and her colleagues (Cheah et al., 2020) have reported disturbing results, based on their survey of 543 Chinese American parents and a subsample of 230 of their children between the ages of 10 and 18 years. Reportedly, 31.7% of the parents and 45.7% of children have been victims of Asiaphobia online (e.g., somebody made anti-Asian remarks specific to COVID-19). Perhaps even more troubling, a majority of the parents (50.9%) and children (50.2%) reported to have directly experienced COVID-19-related Asiaphobia in person, with even higher proportions (88.5% of the parents and 91.9% of the children) reportedly having witnessed the same. These data suggest that experiences of COVID-19-related Asiaphobia are predictive of poor overall psychological health for Asian Americans, including generalized anxiety and depressive symptoms. The results obtained by Cheah and her group, thus, highlight the critical importance of addressing Asiaphobia while planning school reopenings for three major reasons: (1) experiences of Asiaphobia appear to be highly prevalent; (2) experiences of Asiaphobia seem far more pervasive in person than online; and (3) experiences of Asiaphobia have been linked to compromised overall psychological health as well as symptoms of generalized anxiety and depression in children.

Resurgence of COVID-19 infection rates have prompted extended delays in in-person school reopening across the nation and, in some cases, schools that once reopened have been forced into closing again in the U.S. (Mayor De Blasio Holds Media Availability, 2020). As debates over school reopening are expected to continue for the foreseeable future during the pandemic, it would be imperative that educators, policymakers, and other stakeholders develop strategies to eradicate or, at a minimum, alleviate Asiaphobia.

## Mechanisms of Scapegoating Asians

White (2020) notes that, historically, non-White groups have been scapegoated for various infectious diseases and xenophobic reactions have generally been justified as measures to control the spread of infections. One could thus argue that, in the early stages of the outbreak when most infections were contained in China, it would have perhaps made logical sense to be wary of traveling to or from the affected areas, or to limit contact with people with physical ties to such areas (Arnot and Mzezewa, 2020). However, in the current pandemic, the stigma has been associated with anybody that looks Asian, making the target far more generalized (Misra et al., 2020). In addition, the epicenters of infections swiftly shifted from China to Iran and Southern Europe in the spring of 2020, and then to the U.S., India, and Brazil over the summer. Then, a recent resurgence has been prevalent in the U.S., Latin America, and parts of Europe as of late October, 2020 (World Health Organization, 2020c). Interestingly, the same WHO data (2020c) indicate that widespread infections has not transpired in Asian nations such as Japan and Korea, despite their relative geographic proximity to Wuhan, China. Taken together, ostracizing Asians would appear to make little sense as a way to control infection and it may thus be feasible that Asiaphobia in the present context is reflective of other motives, such as affective reactions (see Hsu, 2020).

At the time of writing, no empirical research seems to have been published, systematically investigating the reasons underlying COVID-19-related anti-Asian xenophobia. However, the following may be among the key contributing factors. First, the notion of *envied minority* status (Lin et al., 2016) may provide a potential explanation for Asiaphobia during the pandemic. Consistent with the *model minority* stereotype, Asian Americans as a group have been shown not only to have higher *economic* and *educational* success than other groups in the U.S., including Whites (Nguyen et al., 2020), but also to enjoy better *health* than others (Le et al., 2020). The perceived overall well-being of Asian Americans as a group has been theorized to make Asian Americans an *envied outgroup*, which can lead to dehumanized views of Asians from the non-Asian standpoint. Further, this can prevent non-Asian Americans from individualizing Asians or feeling sympathetic when Asian Americans experience hardships (Lin et al., 2016). Fiske (2010) elaborates that individuals, at times, experience *schadenfreude* and “…*can't help smiling a little”* when bad things happen to members of envied outgroups (p. 703). Accordingly, in the context of the current pandemic, it would be reasonable to hypothesize that, because of the status Asian Americans hold as *envied outgroups*, Asians may particularly be vulnerable to xenophobia and, in fact, non-Asians may show little empathy toward Asians being victimized.

Second, the pandemic is showing little signs of slowing down and, in fact, the infection rates are increasing in many areas across the globe, according to the World Health Organization (2020c) statistics. As of mid-November, 2020, confirmed new cases worldwide are on the rise again (World Health Organization, 2020c), signaling that we may not see the end of the pandemic anytime soon. In addition to these enduring health-related threats, our “new normal” entails widespread *shelter-in-place* orders, economic instability, and extended business and school closures in many parts of the world, likely inducing various negative emotions such as frustration, fear, and anger (Croucher et al., 2020). With 35 million people in the U.S. potentially facing eviction for non-payment of rents or mortgages (Nova, 2020), for instance, a new wave of financial crises may surface in the upcoming months. As Asians have frequently been scapegoated for being responsible for the current pandemic with the virus's presumed origin in China (Misra et al., 2020), it may make logical sense to predict that, without intervention efforts, there may be further spikes of Asiaphobia.

Finally, U.S. government officials continue their sweeping anti-China rhetoric and act in ways that may further normalize, if not encourage, Asiaphobia (Congressional Asian Pacific American Caucus [CAPAC]., 2020) in both adults and children. Repeatedly referring to COVID-19 as the “China Virus” or “Chinese Virus” and arguing the legitimacy of these terms, for example, President Trump and his staff have consistently blamed China for the current pandemic, ostensibly to justify a broader anti-China campaign (Nakamura and Morello, 2020). At times, his anti-Chinese sentiment is conveyed in ways that would appeal to children. For instance, around the age of 3 years, children begin to engage in various language plays, such as rhyming-based creation of non-sensical words (Martin, 2006). President Trump has referred to COVID-19 as “kung flu,” which is a non-sensical word that rhymes with “kung fu” and, given the developmental phases associated the appreciation of word play of this nature, without preventive measures, his “kung flu” reference is likely to encourage Asiaphobia among children. In sum, there seem to be a range of psychological, economic, and sociopolitical dynamics that may make Asian Americans further vulnerable to xenophobia, and it is thus clear that proper measures need to be taken in discussing school reopenings.

## School Reopening in the U.S. and *Asiaphobia*

In the U.S., among the abrupt changes children and their families faced at the onset of the pandemic was that virtually all Kindergarten through Grade 12 (K-12) schools discontinued their in-person classes and converted them online. As of mid-November, 2020, some U.S. schools have resumed in-person classes with modifications (e.g., limiting the number of students that can report to school each day, etc.) while others, such as public schools in Los Angeles and Philadelphia, have remained entirely virtual (Blume, 2020). Yet others (e.g., in New York City) temporarily resumed modified in-person classes, only to revert back to online instruction quickly due to a resurgence in infections (Graham, 2020). This signals the fluid complexities associated with any plans to reopen schools in the midst of the current pandemic, and debates over whether, how, or when K-12 schools should reopen are thus likely to persist for some time (Baker, 2020). With recurring rises in new infection cases across the U.S., skepticism has been voiced by health experts that school reopening needs to be approached with extreme caution, some even suggesting that schools may not reopen safely for in-person classes, even in modified formats or with a rapid development of vaccines, until September, 2021 (Binkley, 2020; Graham, 2020).

Discussions on how best to reopen schools have included “hardware” adjustments, such as installing shields and rearranging furniture to establish safe personal space, as well as “software” adjustments, including frequent cleaning and having children report to school in small groups at a time while others stay home (Binkley, 2020). These conversations have frequently grown contentious and, on the whole, it is likely that plans to safely reopen the nation's schools will continue to be debated well into 2021.

### *Asiaphobia* and the Model Minority Myth in the U.S. Schools

In addition to establishing effective anti-infection measures toward school reopening in the U.S., considering the recent resurgence of anti-Asian hate incidents, schools, educators, and other stakeholders should be mindful of the physical and psychological safety of students of Asian descent. Earlier, a possibility was raised that, perhaps due to the *envied outgroup* status, non-Asians in the U.S. might not consider Asiaphobia particularly problematic. Consistent with this presupposition, American schools have long been less than willing to cater to the needs of Asian students, presumably due to the model minority myth (Poon et al., 2016). Chou and Feagin (2015) explain that, since Asian students in U.S. schools, on average, outperform their non-Asian peers and are comparatively unlikely to display behavioral problems, American schools have historically *deprioritized* the needs of Asian students. This may explain the trend whereby, despite Asian American children being more likely than their counterparts from other backgrounds to be bullied due to their ethnicity, schools frequently fail to take action against these incidents (Wang et al., 2016). This, combined with the data reported by Cheah et al. (2020) that the experience of Asiaphobia is more prevalent in in-person settings than online, would clearly suggest that it would be irresponsible to develop school reopening plans without well-articulated strategies to protect students of Asian descent.

## Discussion: ideas for Interventions

Some have compared the Asiaphobia in the current pandemic to the discrimination faced by Muslims and individuals of Middle Eastern descent after the terror attacks on September 11, 2001 or to the internment of Japanese Americans post-Pearl Harbor in 1941 (Liu and Modir, 2020). There are some monumental differences between COVID-19 and these two incidents (e.g., the pandemic, unlike terror attacks, is sustained over a period of time and the outcomes remain unpredictable, etc.); still, lessons from the past could provide guidance to fight Asiaphobia, as follows, through: (1) condemnation of xenophobia; (2) correcting misinformation; and (3) humanizing Asians as a group.

### Schoolwide Condemnation of Asiaphobia

Anti-Muslim sentiment permeated through the entire nation after the attacks on September 11th, 2001 in the U.S.; in response, just a week after the attacks, President George W. Bush visited the Islamic Center in Washington, D.C. to show his support for the Muslim community. There, President Bush publicly denounced anti-Muslim xenophobia, characterizing individuals engaging in such xenophobic acts as “worst humankind,” successfully raising public awareness to be mindful of the dire need to defuse the anti-Muslim climate (Baer and Greene, 2019). Following this example, it would be reasonable to propose that, by acknowledging Asiaphobia as a pervasive problem Asian American students face and denunciating any act of Asiaphobia, schools could help promote awareness to fight Asiaphobia among children, parents, staff, and faculty.

When Congresswoman Grace Meng of the U.S. House of Representatives sponsored a measure in September, 2020, simply proposing to express the collective condemnation of pandemic-related anti-Asian discrimination, surprisingly, her proposal was met with resistance. Although the measure passed in the end, 40% of the congresspersons voted in opposition (Yam, 2020). The fact that the federal government has been reluctant to condemn Asiaphobia makes it even more critical that schools, led by strong leadership, articulate unequivocal condemnation of anti-Asian xenophobia. Such condemnation may be publicly communicated prior to school reopening through school websites, newsletters, and notes to parents, and the message could be reinforced with children through such settings as school assembly and morning meetings. Provided that schools typically have anti-bullying policies in place, furthermore, discussions on Asiaphobia with children should perhaps draw direct reference to such policies. Additionally, seeking support from local government and community leaders may serve to further strengthen the message denouncing Asiaphobia.

### Fighting Disinformation and Humanizing Asians

In reference to the attacks on September 11, 2001 and the internment of Japanese Americans, educators nationwide have invested a great deal of effort into developing curricula to promote the understanding of facts while breaking down stereotypes and animosity (Miksch and Ghere, 2004). As part of schoolwide efforts to mitigate Asiaphobia, it would be useful to discuss disinformation about COVID-19, correcting it as appropriate. WHO has a webpage dedicated to the reduction of anti-Asian and other social stigma associated with the pandemic (e.g., Asians posing danger to public health; World Health Organization, 2020b), which includes a link to a number of resources that present information categorically refuting a lengthy list of misconceptions, irrational fear, and conspiracy theories surrounding COVID-19 (World Health Organization, 2020a). Such information could serve the dual purposes of defusing Asiaphobia while communicating the correct information to children and their families to promote healthy behavior, and it could be: (i) included with the school communiques discussed above; (ii) during school assembly and morning meetings; or (iii) incorporated into curricula, such as civic classes.

Given the sheer amount and complexity of information being disseminated daily on COVID-19, one may be tempted to resort to using heuristics—or cognitive shortcuts—in processing pandemic-related information. Notoriously, the use of heuristics has been closely linked to stereotypic judgments (Gruber et al., 2020). With references made to China routinely in discussing the pandemic as discussed above, this may create a tendency among non-Asians to put all Asians into one broad category without recognizing the vast within-group variability. Earlier, it was discussed that the envied outgroup status held by Asians may be contributing to Asiaphobia, even potentially prompting some non-Asians to derive pleasure from seeing Asian Americans being victimized. Among the cornerstones of this notion are the dehumanized and deindividualized views of Asians; as such, effort to humanize and individualize Asians should be useful in defusing Asiaphobia.

With 48 countries in Asia, Chinese people merely represent one segment of the Asian community. Furthermore, it should be emphasized that even the Chinese American category is far from homogeneous along an array of domains, from geographic origin, citizenship, language, political orientation, to socioeconomic status (Wu, 2018). Discussions on Asia and its people emphasizing within-group diversity should serve to highlight the irrational nature of painting all Asians with one wide brush, further reinforcing the schoolwide message, condemning Asiaphobia.

## Conclusions

The continued anti-Asian xenophobia and persistent threats of anti-Asian hate incidents in the midst of the pandemic are bound to have psychological consequences among the impacted population, and such evidence is beginning to emerge (Cheah et al., 2020). In fact, actual or anticipated stigma and discrimination experienced by individuals of Asian descent in the current pandemic, according to Misra et al. (2020), are theorized to be particularly damaging because of the extraordinary pandemic-related stress individuals of all backgrounds experience. Thus, schools, educators, and other stakeholders have the responsibility, not only to alleviate anti-Asian stigma and discrimination but also to provide COVID-19-related support for all students, so as to create a climate where academic learning, peer relationship, and psychological well-being are all holistically promoted.

As part of school reopening plans, schools, and educators should widely publicize their condemnation of xenophobia and reiterate their anti-bullying policy. While the protection of students of Asian descent has been the focus here, stigma has been emerging against, among others, frontline workers and their families as well as individuals that have survived COVID-19 (Bagcchi, 2020). Perhaps taking this unfortunate pandemic as a teachable moment, students need to be reminded of the importance of remaining respectful, considerate, and supportive of all peers.

## Data Availability Statement

The original contributions presented in the study are included in the article/supplementary material, further inquiries can be directed to the corresponding author/s.

## Author Contributions

DA is solely responsible for the entire manuscript, from the conceptualization, literature search, and writing to proofreading.

## Conflict of Interest

The author declares that the research was conducted in the absence of any commercial or financial relationships that could be construed as a potential conflict of interest.
